# TNF-α promotes α-synuclein propagation through stimulation of senescence-associated lysosomal exocytosis

**DOI:** 10.1038/s12276-022-00789-x

**Published:** 2022-07-05

**Authors:** Eun-Jin Bae, Minsun Choi, Jeong Tae Kim, Dong-Kyu Kim, Min Kyo Jung, Changyoun Kim, Tae-Kyung Kim, Jun Sung Lee, Byung Chul Jung, Soo Jean Shin, Ka Hyun Rhee, Seung-Jae Lee

**Affiliations:** 1 Neuroscience Research Institute, Seoul National University College of Medicine, Seoul, 03080 Korea; 2 Department of Biomedical Sciences, Seoul National University College of Medicine, Seoul, 03080 Republic of Korea; 3 Neural Circuits Research Group, Korea Brain Research Institute, Daegu, 41068 Korea; 4 Department of Exercise Physiology and Sport Science Institute, Korea National Sport University, Seoul, 05541 Republic of Korea; 5 Present Address: Center for Convergence Research of Neurological Disorders, Ajou University School of Medicine, Suwon, 16499 Korea; 6 Present Address: Molecular Neuropathology Section, Laboratory of Neurogenetics, National Institute on Aging, National Institutes of Health, Bethesda, MD 20892 USA; 7 Present Address: Neuramedy Co., Ltd., Seoul, Korea; 8 Present Address: Nutritional Sciences and Toxicology Department, University of California Berkeley, Berkeley, CA 94720 USA

**Keywords:** Parkinson's disease, Neurodegeneration, Cellular neuroscience

## Abstract

Cell-to-cell propagation of α-synuclein is thought to be the underlying mechanism of Parkinson’s disease progression. Recent evidence suggests that inflammation plays an important role in the propagation of protein aggregates. However, the mechanism by which inflammation regulates the propagation of aggregates remains unknown. Here, using in vitro cultures, we found that soluble factors secreted from activated microglia promote cell-to-cell propagation of α-synuclein and further showed that among these soluble factors, TNF-α had the most robust stimulatory activity. Treatment of neurons with TNF-α triggered cellular senescence, as shown by transcriptomic analyses demonstrating induction of senescence-associated genes and immunoanalysis of senescence phenotype marker proteins. Interestingly, secretion of α-synuclein was increased in senescent neurons, reflecting acquisition of a senescence-associated secretory phenotype (SASP). Using vacuolin-1, an inhibitor of lysosomal exocytosis, and RNAi against *rab27a*, we demonstrated that the SASP was mediated by lysosomal exocytosis. Correlative light and electron microscopy and immunoelectron microscopy confirmed that propagating α-synuclein aggregates were present in electron-dense lysosome-like compartments. TNF-α promoted the SASP through stimulation of lysosomal exocytosis, thereby increasing the secretion of α-synuclein. Collectively, these results suggest that TNF-α is the major inflammatory factor that drives cell-to-cell propagation of α-synuclein by promoting the SASP and subsequent secretion of α-synuclein.

## Parkinson’s disease: A vicious cycle of inflammation and protein aggregation

A signaling protein known as a cytokine produced during inflammation promotes the spread of protein aggregates that cause Parkinson’s disease. Several studies have suggested a role for inflammation in forming aggregates of the neuronal protein α-synuclein, the pathological hallmark of Parkinson’s disease, but the exact mechanisms remain unclear. Now, Seung-Jae Lee at Seoul National University College of Medicine, South Korea, and co-workers have revealed a key role for an inflammatory cytokine called TNF-α. The team showed that TNF-α triggers cellular senescence, where cells undergo aging process, thereby stimulating the exocytosis mediated by lysosomes, or waste disposal system, in neurons. Over time, this results in an increased number of senescent cells, which secrete more α-synuclein than normal. The increased α-synuclein levels can in turn activate immune cells to sustain inflammation, setting up a feedback cycle to keep the disease progressing.

## Introduction

Parkinson’s disease (PD), characterized by complex motor symptoms caused by selective degeneration of dopaminergic neurons in the substantia nigra pars compacta (SNpc), is the second-most common age-related neurodegenerative disorder. Progressive accumulation of α-synuclein-enriched inclusion bodies, called Lewy bodies and Lewy neurites, is the pathological hallmark of PD^1^. Multiplication and missense mutations in *SNCA*, the gene encoding α-synuclein, have been linked to autosomal-dominant forms of familial PD^2,3^. Genome-wide association studies have further shown that *SNCA* is associated with sporadic forms of PD^4^. These genetic and pathological studies have indicated that the aggregation of α-synuclein is the major contributor to the pathogenesis of PD.

PD is a progressive disease with various symptoms, including autonomic and sensory dysfunctions, rapid eye movement, sleep behavior disorder, dementia, and hallucinations, that develop over the course of the disease^5^. The currently dominant explanatory hypothesis is that symptomatic progression is driven by the spread of α-synuclein aggregates, which initially occur in lower brainstem nuclei and the olfactory bulb, progressively spread through the midbrain and mesocortex, and finally affect neocortical regions^6–9^. The challenge is to understand the mechanism of aggregate spreading and leverage this insight to develop strategies for stopping the progression of the disease.

A large body of evidence supports the idea that cell-to-cell propagation of α-synuclein aggregates is the underlying mechanism for spreading of α-synuclein pathology^10^. One previous study reported that α-synuclein aggregates spread via exocytosis and subsequent endocytosis of the aggregates^11^, whereas another suggested that the propagation is mediated by tunneling nanotubes^12^. Exocytosis of α-synuclein aggregates appears to be a regulated process that is activated under stress conditions^13–15^. However, the exocytosis mechanism remains unknown.

Another pathological feature of PD in addition to the aggregation of α-synuclein is neuroinflammation. Analyses of postmortem brain tissues from patients with PD have demonstrated elevation of a number of inflammatory features, including “activated” microglia and astrocytes, proinflammatory cytokines, and the NLR family, pyrin domain containing 3 (NLRP3) inflammasome^16–18^. Studies have shown that inflammation promotes the formation of protein aggregates. For example, injection of the inflammagen lipopolysaccharide (LPS) caused α-synuclein aggregation as well as dopaminergic cell death in mice^19^. Our recent study showed that injection of α-synuclein multimers into the striatum leads to inflammatory responses prior to the spread of α-synuclein aggregates and that treatment with an anti-inflammatory agent prevents α-synuclein pathology (see related manuscript file). These observations are consistent with the earlier finding that inflammation precedes α-synuclein aggregation in grafted mesencephalic tissues in the brains of PD patients^20^. These results suggest that an inflammatory microenvironment promotes pathological α-synuclein aggregation and propagation in the brain and thereby contributes to disease progression. However, the mechanism by which an inflammatory microenvironment promotes α-synuclein aggregation remains unclear.

In the present study, we found that α-synuclein is secreted from cells via the senescence-associated secretory phenotype (SASP), which in turn is mediated by lysosomal exocytosis. We also show that the proinflammatory cytokine TNF-α promotes neuronal senescence and the development of the SASP, thereby promoting α-synuclein secretion and propagation.

## Materials and methods

### Cell culture

Human neuroblastoma SH-SY5Y cells were cultured and differentiated as described previously^21^. For knockdown of RAB27a, cells were transduced with RAB27a shRNA (Santa Cruz Biotechnology, Inc., Dallas, TX, USA, sc-41834-SH) according to the manufacturer’s instructions.

### Cell-to-cell propagation assay

Cell-to-cell propagation of α-synuclein was assessed as previously described^21^ using the dual-cell bimolecular fluorescence complementation (BiFC) coculture system. In some experiments, microglial conditioned media were added to V1S and SV2 cocultures, and cells were cultured for two additional days. For treatment with recombinant inflammatory cytokines, V1S and SV2 cells were cocultured for 3 days and then treated with human recombinant cytokines for the last 24 h. For fluorescence microscopic analysis, five microscope fields for each sample were selected at random; an average of 100 cells were analyzed per sample. The human recombinant cytokines used in this assay are listed in Supplementary Table 2.

### Primary microglial culture

Primary microglia were cultured as previously described^22^. Briefly, primary microglial cells were obtained by digestion of the cerebral cortex of a 1-day-old neonatal C57BL/6 mouse with trypsin–EDTA at 37 °C. After treatment with DNase I (Roche, Basel, Switzerland, #11284932001), the cells were centrifuged at 500 × *g* for 5 min and resuspended in DMEM/F12 containing 10% fetal bovine serum (FBS). Cells were filtered through a 120-μM nylon mesh filter, plated onto poly-D-lysine-coated dishes at 37 °C, and maintained in DMEM/F12 containing 10% FBS, with medium replacement every 3–4 days. On Day 10, microglia were detached by gently tapping the dishes and then collected by centrifugation. The resulting microglial cell pellets were resuspended, plated onto poly-D-lysine-coated wells, and used for experiments the next day.

### Primary cortical neuron culture

Primary cortical neurons obtained from embryonic Day 16 (E16) embryos of a pregnant C57BL/6 mouse or Sprague-Dawley rat were cultured as previously described^23^. Briefly, cerebral cortices were dissected and incubated with papain solution (HBSS containing 10 U/ml papain, 0.2 mg/ml cysteine, 0.5 mM EDTA, 1 mM CaCl_2_, and 0.003 N NaOH) for 20 min, followed by the addition of DNase I (Roche, #11284932001). Tissues were dissociated mechanically by pipetting in culture medium and then centrifuged briefly to collect the cells. Cells were plated on poly-D-lysine/laminin-coated plastic dishes or poly-D-lysine/Matrigel-coated (BD Biosciences, San Diego, CA, USA) glass coverslips. The culture medium was changed every 3 days.

### Preparation of conditioned medium

Primary microglial cells were plated in 60-mm culture dishes, and after incubating overnight, they were treated with LPS (100 ng/ml) or vehicle control (DMSO) with or without inhibitors of the transcription factors (1 μM) for 1 h. Microglia were then washed with fresh media and incubated for an additional 4 h. The several transcription factor inhibitors used in this experiment are as follows: NF-κB (Bay 11-7085; Tocris, Ellisville, MI, #S7352), AP-1 (SR 11302; Tocris, #2476), or the LPS inhibitor (Polymyxin B; Sigma-Aldrich, St. Louis, MO, USA, #81334).

### Reverse transcription-quantitative PCR (RT-qPCR)

Total RNA was extracted from primary microglial cells and primary neurons using an RNeasy Mini Kit (Qiagen, Hilden, NRW, Germany, #74106), and cDNA was synthesized by reverse transcription using the iScript cDNA synthesis kit (Bio-Rad, Hercules, CA, USA, #1708891) according to the manufacturer’s instructions. Target genes were amplified using iTaq Universal SYBR Green Supermix (Bio-Rad, #172-5121) with specific primers. Primer sequences are listed in Supplementary Table 3. The thermocycling program for mouse samples consisted of 95 °C for 30 s, followed by 40 cycles of 95 °C for 5 s and 58 °C for 30 s using the CFX Connect Real-time PCR system (Bio-Rad, #1855201). Nontemplate controls were included for each set of primers. Relative mRNA levels were calculated according to the 2^−ΔΔCT^ method. All ΔCτ values were normalized to those of glyceraldehyde-3-phosphate dehydrogenase (GAPDH).

### Western blotting

Western blotting was performed as previously described^22^. Blots were imaged using an Amersham Imager 600 (GE Healthcare, Chicago, IL) and Multi Gauge (v.3.0) software (Fujifilm, Akishima, Tokyo, Japan). The antibodies used in this study are listed in Supplementary Table 4.

### Senescence-associated β-galactosidase (SA-β-gal) activity

SA-β-gal is detectable using the β-galactosidase substrate C12FDG. Primary cortical neurons were plated on poly-D-lysine (0.1 mg/ml; Sigma-Aldrich, #P7280)- and Matrigel (1:100; BD Biosciences)-coated coverslips and incubated overnight. The next day, the cells were first incubated with bafilomycin A1 (0.1 nM) for 1 h at 37 °C and then treated with 33 μM C12FDG dissolved in prewarmed fresh culture media for 2 h at 37 °C.

### Immunofluorescence staining

Immunofluorescence staining was performed as previously described^21^ using the antibodies listed in Supplementary Table 4. After immunostaining, slide glass-plated cells were counterstained with 4’,6-diamidino-2-phenylindole (DAPI; Invitrogen, Carlsbad, CA, D1306) and coverslip-mounted in the presence of Prolong Gold Antifade Reagent (P36930, Invitrogen). Images were acquired using a Zeiss LSM 700 confocal laser-scanning microscope.

### Animals

B6;129S-Tnftm1Gkl/J mice, purchased from The Jackson Laboratory (Bar Harbor, ME, USA, #003008), were housed at the animal facility of the Seoul National University College of Medicine under standardized conditions. All mice were habituated in the animal facility for at least 1 week before the start of experimentation.

### Generation of recombinant α-synuclein multimers

Recombinant human α-synuclein V40G was purified as previously described^14^. For fibrillation, α-synuclein (200 μM in PBS) was incubated at 37 °C for 9 days with constant shaking at 1050 rpm.

### Stereotaxic injection of α-synuclein V40G multimers

For intrastriatal injection of α-synuclein V40G multimers or phosphate-buffered saline (vehicle control), 10-week-old male C57BL/6 mice were anesthetized with ketamine hydrochloride and xylazine hydrochloride (3.5:1, 2.5 µl/g). PBS or V40G multimers (6 µg) in a volume of 2 µl were stereotaxically injected into the right striatum (anterior/posterior, 1.0 mm; medial/lateral, 1.5 mm; and dorsal/ventral, 3.0 mm) at a rate of 0.5 μl/min using a 30 G needle.

### Sample collection

Twenty weeks after intrastriatal injection, mice were anesthetized with 1.2% Avertin (0.23 ml/g) and then transcardially perfused with saline followed by ice-cold 4% paraformaldehyde (PFA). Brains were dissected out and fixed in 4% PFA in PBS for at least 48 h at 4 °C. After fixation, the brain was washed with PBS, and free-floating sections (40-μm thick) were prepared using a Vibratome (Leica Biosystems, Germany, VT1000S).

### Immunohistochemistry and neuropathological analysis

Free-floating brain sections (40-μm thick) were blocked with 4% bovine serum albumin in PBS containing 0.1% Triton X-100 (PBST) and then incubated overnight at 4 °C with primary antibodies. The sections were washed with PBST, incubated with secondary antibodies diluted in PBST, and detected using avidin-biotin-peroxidase complex (ABC Elite kit; Vector Laboratories, Burlingame, CA, USA, #PK6200). Thereafter, 3,3-diaminobenzidine (DAB)-stained sections were imaged using a Zeiss AX10 microscope (Carl Zeiss, Germany). The level of immunoreactivity against phospho-α-synuclein was determined by optical density analysis using ImageJ (NIH) and corrected against background signal levels.

### Strain and culturing of worms

The experiments used three representative lines of BiFC transgenic worms, generated as previously described^24^. All worms were grown and maintained on nematode growth medium (NGM) plates seeded with *Escherichia coli* strain OP50 at 20 °C in accordance with standard procedures.

### RNA interference (RNAi) in *C. elegans*

RNAi analyses were performed based on a previously described bacterial feeding protocol^25^. L4-stage worms were transferred to an NGM plate containing 25 μg/ml ampicillin (Sigma-Aldrich, A9518) and 1 mM isopropyl-1-thio-β-D-galactopyranoside (IPTG; Beams Biotechnology, Seongnam, Korea, #7001), fed bacteria expressing dsRNA against *rab-27*, and then incubated at 20 °C. RNAi-expressing bacterial clones were a generous gift from Professor Ao-Lin Hsu (National Yang-Ming University, Taipei, Taiwan). After we obtained F1 progeny produced from worms in which dsRNA-expressing bacteria were introduced, the worms were washed in M9 buffer (22 mM KH_2_PO_4_, 22 mM Na_2_HPO_4_, 85 mM NaCl, 1 mM MgSO_4_), immobilized with 10 mM sodium azide (Sigma-Aldrich, S2002) in M9 buffer, and then placed onto a 96-well plate (Corning, #3904). Images of worms were acquired using an IN Cell Analyzer 2000 system (Cytiva, Marlborough, MA, USA), and fluorescence intensity was quantified using IN Cell Investigator Developer software (Cytiva).

### RNA processing

RNA was extracted using an RNeasy Mini Kit (Qiagen). Libraries were prepared using a QuantSeq 3’ mRNA-Seq Library Prep Kit (Lexogen, Inc., Greenland, NH, USA). cDNA was sequenced with 75-bp single-end reads on a NextSeq 500 sequencing system (Illumina, Inc., San Diego, CA, USA).

### RNA sequencing (RNA-seq) analysis

RNA sequencing was performed using TopHat2 (version 2.1.1), and data were processed and quantified using HTSeq. Analyses of differentially expressed genes (DEGs; fold change >0.3 and *p* value <0.05) were performed using DESeq2 (version 1.24.0)^26^. Gene Ontology (GO) enrichment analyses were performed using the Cytoscape plug-in ClueGO^27^. Gene set enrichment analysis (GSEA) was performed using GSEA Java application GSEA_4.1.0 (Broad Institute and University of California San Diego). GO enrichment and Kyoto Encyclopedia of Genes and Genomes (KEGG) pathway analyses of DEGs were performed on DAVID datasets (version 6.8)^28^.

### Enzyme-linked immunosorbent assay (ELISA)

The procedures for sandwich ELISAs have been previously described in detail^29^. Freshly prepared serial dilutions of mouse recombinant α-synuclein monomer were loaded as standards. The antibodies used in this study are listed in Supplementary Table 4. TNF-α levels were measured using a Mouse TNF-alpha Quantikine ELISA Kit (SMTA00B; R&D Systems, Minneapolis, MN, USA) according to the manufacturer’s protocols.

### Correlative light and electron microscopy (CLEM)

V1S and SV2 cells were grown to 50–60% confluence in 35-mm glass-bottomed culture dishes. Cells were imaged under a confocal light microscope (LSM780; Carl Zeiss). Cells were fixed by incubating with sodium cacodylate buffer containing 2% paraformaldehyde (EM grade; EMS) and 2.5% glutaraldehyde (EMS). After washing, the cells were postfixed in 2% osmium tetroxide (OsO_4_) containing 1.5% potassium ferrocyanide for 1 h at 4 °C, dehydrated using an ethanol series (50%, 60%, 70%, 80%, 90%, and 100%; 10 min each) and infiltrated with embedding medium. After embedding, the target of interest was identified by applying numbers and letters to the surface of the sample containing the cells. Identified samples were cut into 60-nm sections horizontal to the plane of the block (UC7; Leica Microsystems), mounted onto copper slot grids with a specimen support film, and then double-stained with 2% uranyl acetate (10 min) and lead citrate (5 min). The sections were then examined using a Talos L120C transmission electron microscope operating at 120 kV (Thermo Fisher, Waltham, MA).

### Immunogold label electron microscopy

For immunogold label electron microscopy, cells were fixed by incubating in 2.5% glutaraldehyde and 2% paraformaldehyde in sodium cacodylate buffer (pH 7.2) at 4 °C. The specimens were then fixed in 0.5% osmium tetroxide (OsO_4_) for 30 min at 4 °C, dehydrated using an ethanol gradient (50%, 60%, 70%, 80%, 90%, and 100%; 20 min each), and transferred to EM812 medium (EMS). After impregnation with pure resin, the specimens were embedded in the same resin mixture. The samples were sectioned (60 nm) with an ultramicrotome (UC7; Leica Microsystems) and then collected on nickel grids. Postembedding immunogold labeling was performed using primary antibodies and 9–11 nm colloidal gold-conjugated anti-mouse (Sigma-Aldrich, #G7652) or 5 nm colloidal gold-conjugated goat anti-rabbit (Sigma-Aldrich, #G7277) IgG secondary antibodies, as appropriate. Following immunogold labeling, sections were double-stained with 2% uranyl acetate (10 min) and lead citrate (5 min). Sections were then examined using a transmission electron microscope operating at 120 kV (Tecnai G2; Thermo Fisher).

### Statistical analysis

The statistical significance of differences among groups was evaluated using a two-tailed unpaired Student’s *t* test, one-way analysis of variance (ANOVA) with Dunnett’s post hoc test and Tukey’s post hoc test, or two-way ANOVA with Bonferroni’s post hoc test and Tukey’s post hoc test using GraphPad Prism 9.0.2 (GraphPad Software, Inc., La Jolla, CA, USA).

## Results

### Activated microglia produce soluble factors that promote cell-to-cell propagation of α-synuclein

**Fig. 1:**
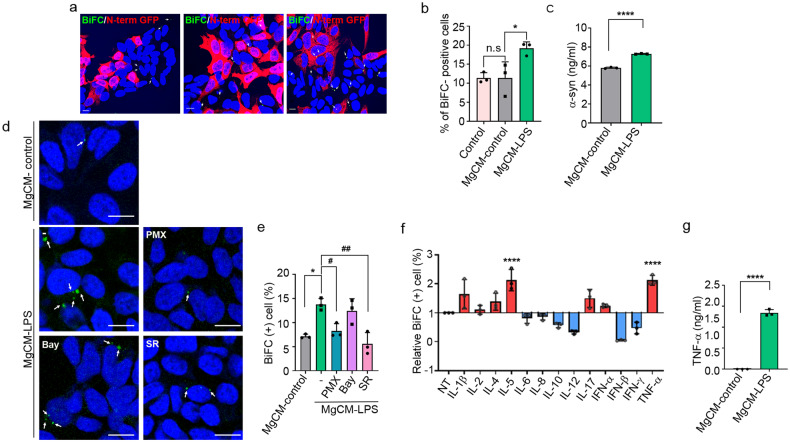
Soluble factors secreted from activated microglia increase the propagation of α-synuclein. **a**, **b** Effects of activated microglia on the cell-to-cell propagation of α-synuclein. BiFC-positive cells are indicated with arrows. Scale bar: 10 μm. **b** Quantification of the percentage of BiFC-positive cells in (**a**). **c** Levels of neuron-secreted α-synuclein. The amount of released α-synuclein was determined by sandwich ELISAs. **d**, **e** Effects of transcription factor inhibitors on propagation-promoting activity. BiFC-positive cells are indicated with arrows. Scale bar: 20 µm. **e** Quantification of the percentage of BiFC-positive cells in (**d**). Bay, Bay 11-7085; SR, SR11302; PMX, polymyxin B. **f** Screening of inflammatory factors (50 ng/ml) for propagation-promoting activity. **g** Levels of secreted TNF-α in cultures of the microglia treated with LPS or vehicle, measured by ELISAs. All data are presented as the mean ± SEM (*P < 0.05, ^*#*^*P* < 0.05, **P < 0.005, ^*##*^*P* < 0.005, ****P < 0.0001). Statistical significance was determined by one-way ANOVA with Tukey’s post hoc comparison between groups (**b**, **e**) and Dunnett’s post hoc comparison between groups (**f**) or by two-tailed unpaired Student’s *t* test (**c**, **g**).

To investigate the effects of activated microglia on the propagation of α-synuclein, we obtained conditioned media from microglial cultures (MgCM) treated with LPS (MgCM-LPS) or DMSO (MgCM-control) and added them to the dual-cell bimolecular fluorescence complementation (BiFC) propagation assay system. In this system, cell-to-cell α-synuclein propagation is monitored by measuring the number of cells exhibiting reconstituted Venus fluorescence^21^. Treatment with MgCM-LPS significantly increased the number of BiFC-positive cells compared with that of the cells treated with MgCM-control, indicating increased propagation of α-synuclein (Fig. 1a, b). To determine the basis for this effect of activated microglia, we measured the levels of α-synuclein in the culture media and found that treatment with MgCM-LPS increased the secretion of α-synuclein (Fig. 1c). These results suggest that soluble factors secreted from activated microglia promote cell-to-cell propagation of α-synuclein by increasing its secretion.

To identify the soluble factors that regulate the propagation of α-synuclein, we obtained conditioned media from microglia cultured with inhibitors of major transcription factors, including Bay 11-7085, an inhibitor of the transcription factor NF-κB, and SR 11302, an inhibitor of activator protein-1 (AP-1). Of these two inhibitors, SR 11302 abolished the effects of MgCM-LPS on the propagation of α-synuclein (Fig. 1d, e), suggesting that the propagation-promoting soluble factors are regulated by AP-1. Polymyxin B (PMX), an inhibitor of LPS, reversed the effects of LPS on microglial production/release of soluble factors, as expected, validating the fidelity of the assay.

Next, we performed a literature search for secreted factors whose expression depends on AP-1, identifying IL-1β, -2, -4, -5, -6, -7, -10, -12, and -17, interferon (IFN)-α, -β, and -γ, and TNF-α as candidates^30–36^. We then screened these factors for propagation-promoting activity and found that TNF-α was the most effective (Fig. 1f). To confirm the production of TNF-α by LPS-treated microglia, we performed ELISAs on microglial culture media. LPS treatment caused a dramatic increase in the levels of secreted TNF-α, indicating that LPS induced robust production of TNF-α by microglia (Fig. 1g).

### TNF-α promotes cell-to-cell propagation and secretion of α-synuclein

**Fig. 2:**
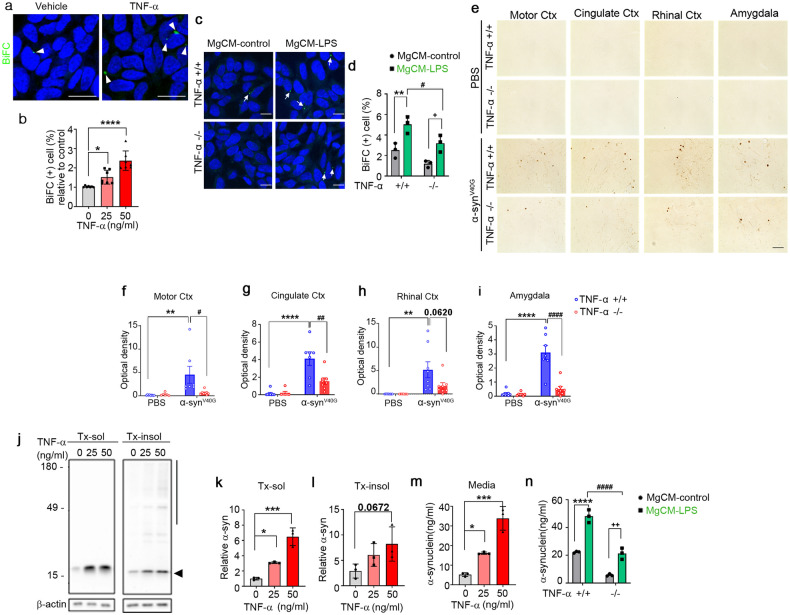
TNF-α regulates α-synuclein propagation. **a**, **b** Effects of TNF-α on the cell-to-cell propagation of α-synuclein. **a** Representative images in which α-synuclein propagation was observed (treated dose: 50 ng/ml). BiFC-positive cells are indicated with arrows. Scale bar: 20 µm. **b** Quantification of the relative percentages of BiFC-positive cells. **c**, **d** Effects of MgCM from cultures of the WT (TNF-α^+/+^) or TNF-α^−/−^ microglia treated with LPS (MgCM-LPS) or DMSO (MgCM-control) on the propagation of α-synuclein. Arrows: BiFC-positive puncta. Scale bar: 20 µm. **d** Percentage of BiFC-positive cells in (**c**). **e**–**i** Effects of TNF-α on the spreading of α-synuclein in vivo. **e** Representative images of phosphorylated α-synuclein (pS129) staining in mouse brain sections at 20 weeks post-injection of α-synuclein^V40G^ multimers (or PBS) into the WT (TNF-α^+/+^) or TNF-α^−/−^ mice. Scale bar: 100 μm. **f**–**i** Levels of pS129. Optical densities were measured in the motor cortex (**f**; WT + PBS, *n* = 8; KO + PBS, *n* = 6; WT + α-synuclein, *n* = 7; KO + α-synuclein, *n* = 8), cingulate cortex (**g**; WT + PBS, *n* = 8; KO + PBS, *n* = 6; WT + α-synuclein, *n* = 7; KO + α-synuclein, *n* = 8), rhinal cortex (**h**; WT + PBS, *n* = 8; KO + PBS, *n* = 7; WT + α-synuclein, *n* = 8; KO + α-synuclein, *n* = 8), and amygdala (**i**; WT + PBS, *n* = 8; KO + PBS, *n* = 7; WT + α-synuclein, *n* = 7; KO + α-synuclein, *n* = 8). **j**–**l** Effects of TNF-α on the accumulation of α-synuclein in neurons. **j** Representative western blot images of α-synuclein in Triton X-100-soluble (Tx-sol) and Triton X-100-insoluble (Tx-insol) fractions. The quantified region is indicated on the right side of the blot. The arrowhead indicates quantified α-synuclein in Tx-sol. The line includes the quantified area in Tx-insol. Quantification of α-synuclein in Tx-sol (**k**) and Tx-insol (**l**) fractions. **m**, **n** Effects of TNF-α on the secretion of α-synuclein in neurons. **m** Secreted α-synuclein in media, measured by ELISAs. **n** Effects of TNF-α-deficient microglia on the neuronal secretion of α-synuclein. Primary neurons were treated with conditioned media acquired from the WT or TNF-α-deficient microglia pretreated with LPS or vehicle. Data are expressed as the mean ± SEM (*P < 0.05, ^*#*^*P* < 0.05 **P < 0.005, ^*##*^*P* < 0.005, ***P < 0.0005, ****P *<* 0.0001, ^*####*^*P* < 0.0001). Statistical significance was determined by one-way ANOVA with Dunnett’s post hoc comparison between groups (**b**, **k**–**m**), two-way ANOVA with Bonferroni’s post hoc comparison between groups (**d**), and two-way ANOVA with Tukey’s post hoc comparison between groups (**f**–**i**, **n**).

To examine the effects of TNF-α on cell-to-cell propagation of α-synuclein, we treated the dual-cell BiFC system with increasing concentrations of TNF-α. Quantification of BiFC-positive cells showed that TNF-α increased the propagation of α-synuclein in a concentration-dependent manner (Fig. 2a, b). Next, to further validate the effects of TNF-α on α-synuclein propagation, we obtained conditioned media from wild-type (WT) microglia (TNF-α^+/+^) and TNF-α-knockout microglia (TNF-α^−/−^) after treatment with LPS or vehicle. We then treated the dual-cell BiFC system with these conditioned media and assayed propagation. As shown in (Fig. 2c, d), propagation-promoting activity was significantly decreased in conditioned media from the TNF-α-deficient microglia, supporting the contribution of TNF-α to the microglia-derived propagation-promoting activity. To further validate the effects of TNF-α on α-synuclein propagation in vivo, we injected multimers of the V40G variant α-synuclein into the striatum of the WT or TNF-α^−/−^ mice. We chose V40G α-synuclein multimers for these experiments because our previous study showed that these proteins produce more robust α-synuclein pathology than preformed WT α-synuclein fibrils (see related manuscript file). At 20 weeks post-injection, we performed an immunohistopathological analysis of brain sections using an antibody against phosphorylated α-synuclein (pS129). Consistent with this previous study (see related manuscript file), pS129 immunoreactivity was increased in various brain regions, including the motor cortex, cingulate cortex, rhinal cortex, and amygdala, of the V40G-α-synuclein-injected WT mice, whereas virtually no pS129 immunoreactivity was detected in the brains of the WT mice injected with vehicle (Fig. 2e–i). Notably, pS129 immunoreactivity was significantly reduced in all these brain regions of the V40G-α-synuclein-injected TNF-α^−/−^ mice (Fig. 2e–i). Collectively, these in vitro and in vivo results suggest that TNF-α promotes the propagation of α-synuclein.

Next, we examined the effects of TNF-α on the secretion of α-synuclein by primary neurons. Treatment of neurons with increasing concentrations of TNF-α resulted in elevated levels of α-synuclein in both cell extracts and culture media (Fig. 2j–m). Secretion of α-synuclein was also increased by treatment of neurons with MgCM-LPS from the WT microglia. This increase was dampened in the neurons treated with MgCM-LPS from the TNF-α-deficient microglia (Fig. 2n). These results suggest that microglia-derived TNF-α increases the secretion of α-synuclein from neurons, thereby promoting cell-to-cell α-synuclein propagation.

### TNF-α induces neuronal senescence

**Fig. 3:**
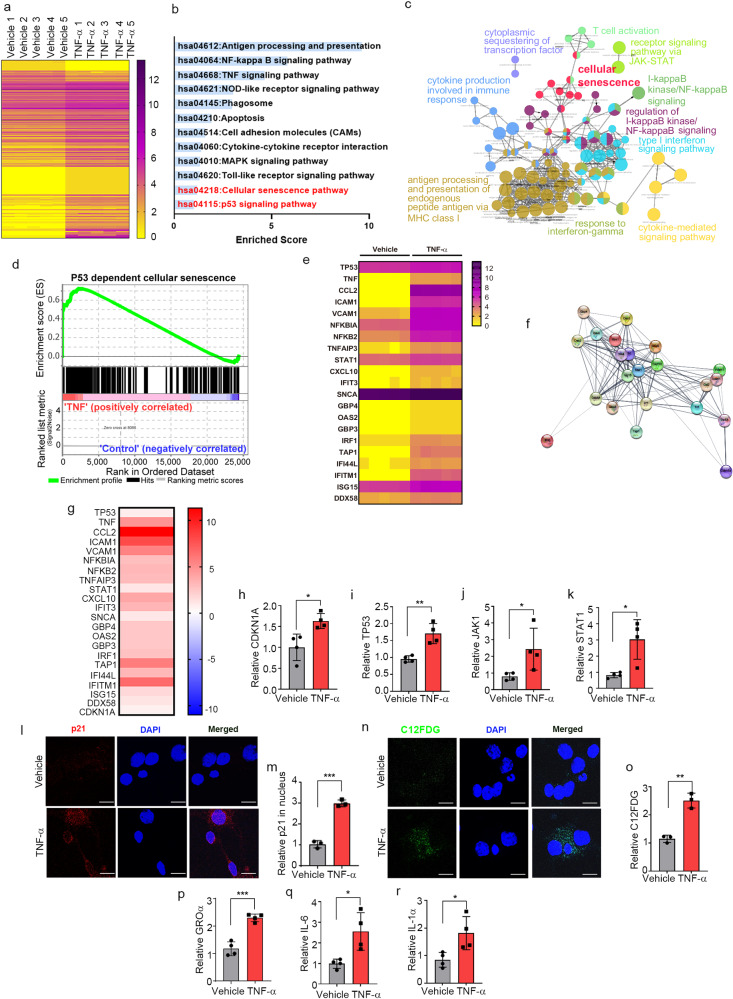
TNF-α induces neuronal senescence. **a** Heatmap representing the expression levels (log2 read count number) of DEGs with upregulated (fold change >1.3) or downregulated (fold change <0.7) expression after treatment with TNF-α or vehicle (*n* = 5 per group). **b** The top 12 enriched KEGG terms for the 118 DEGs in the TNF-α-treated neurons. **c** Simplified networks of significantly enriched GO terms. Each term is statistically significant (Benjamini–Hochberg correction <0.05). The nodes (colored circles) display significantly enriched parent GO terms; the edges show the overlapping genes between terms; and the size of the node represents the number of enriched genes. **d** Enrichment plot of DEGs in the TNF-α-treated *versus* vehicle-treated neurons (FDR *q*-value <0.005). **e** Heatmap of the expression levels of 22 DEGs related to the JAK-STAT senescence pathway. **f** High confidence protein–protein interaction network of the 22 DEGs related to the JAK-STAT senescence pathway, constructed using STRING. **g** Heatmap of the log2-fold changes of the 22 DEGs related to the JAK-STAT senescence pathway. **h**–**k** Expression of *CDKN1A* (**h**), *TP53* (**i**), *JAK1* (**j**), and *STAT1* (**k**), measured by RT-qPCR. **l** Immunofluorescence analysis of p21 protein in primary neurons treated with TNF-α or vehicle. **m** Quantification of relative p21 fluorescence in the nucleus. **n** C12FDG staining representing the accumulation of SA-β-gal. Nuclei were stained with DAPI (blue). **o** Quantification of C12FDG levels. **p**–**r** SASP genes GROα (**p**), IL-6 (**q**), and IL-1α (**r**), measured by RT-qPCR. All data are presented as the mean ± SEM (*P < 0.05, **P < 0.005, ***P < 0.0005). Statistical significance was determined by two-tailed unpaired Student’s *t* test (**h**–**k**, **m**, **o**, **p–r**).

To gain insights into the mechanism by which TNF-α promotes secretion of α-synuclein in neurons, we conducted transcriptomic analyses of neuronal cell following treatment with either vehicle or TNF-α. A total of 24,424 genes were identified after removal of unannotated and duplicated genes. Among them, 118 were identified as differentially expressed genes (DEGs) (Fig. 3a) based on the magnitude of their difference in expression (fold change >0.3) and adjusted *p* values (<0.05) (Supplementary Table 1). Gene Ontology (GO) functional enrichment and Kyoto Encyclopedia of Genes and Genomes (KEGG) pathway analyses revealed that DEGs were involved in cellular senescence as well as apoptotic processes and immune responses, such as antigen processing and presentation, TNF-α signaling, and Toll-like receptor signaling (Fig. 3b, c). Consistent with the results of functional enrichment analyses, gene set enrichment analysis (GSEA) confirmed that DEGs were highly associated with p53-dependent cellular senescence (Fig. 3d). Moreover, gene expression profiling revealed that 22 DEGs were involved in the JAK/STAT-mediated senescence pathway (Fig. 3e–g). To further validate the results of transcriptome analysis, we verified several genes (p21, p53, JAK1, and STAT1) associated with the JAK/STAT-mediated cellular senescence pathway by quantitative reverse transcription-polymerase chain reaction (RT-qPCR), which showed that the expression of these genes was significantly increased in the neurons treated with TNF-α (Fig. 3h–k).

To extend our analysis of TNF-α-induced senescence responses, we treated primary neurons with TNF-α and assessed the expression of cellular senescence marker proteins. Senescent cells undergo morphological changes, chromatin remodeling, metabolic reprogramming, and p21^CIP1/WAF1^ alterations; they also secrete a complex mix of mostly proinflammatory factors, reflecting the acquisition of what is referred to as the senescence-associated secretory phenotype (SASP)^37^. Accordingly, we examined nuclear p21^CIP/WAF1^ expression levels, senescence-associated β-galactosidase (SA-β-gal) activity, and induction of SASP genes as senescence markers in the TNF-α-treated neurons. Nuclear expression levels of p21^CIP/WAF1^ were increased in the neurons treated with TNF-α (Fig. 3l, m), a finding consistent with a previous report that elevated expression and nuclear localization of p21^CIP1/WAF1^ are common hallmarks of aging in postmitotic neurons^38^. Increased SA-β-gal activity, another marker of senescent cells, was detected by monitoring the fluorescence intensity of the fluorogenic SA-β-gal substrate 5-dodecanoylaminofluorescein di-β-D-galactopyranoside (C12FDG). C12FDG fluorescence intensity was significantly increased in the neurons treated with TNF-α compared with the vehicle-treated neurons (Fig. 3n, o). Moreover, the expression levels of the SASP genes GRO*a*, IL-6, and IL-1α were also increased by TNF-α treatment (Fig. 3p–r). Collectively, these results indicate that neurons exposed to TNF-α undergo cellular senescence.

### α-Synuclein is secreted as part of the SASP during neuronal senescence

**Fig. 4:**
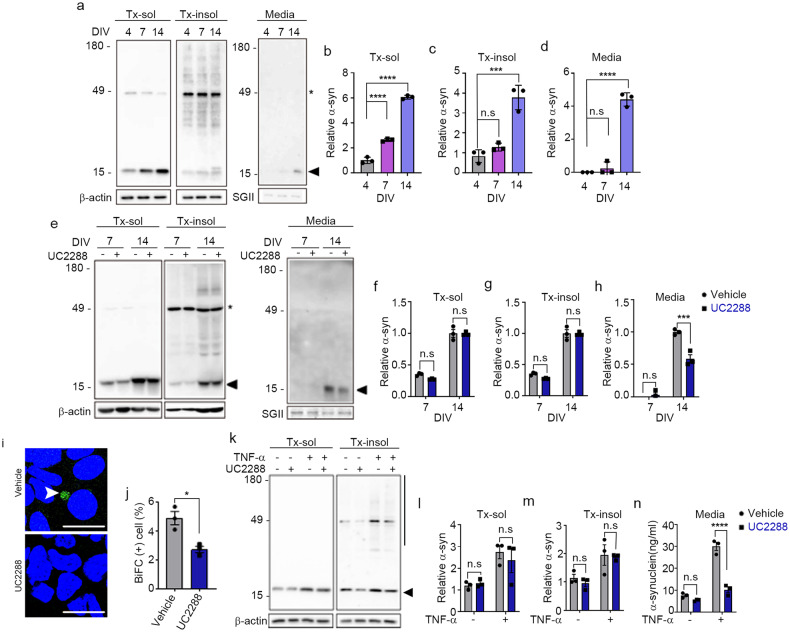
Neuronal senescence modulates α-synuclein propagation. **a**–**d** Secretion of α-synuclein during neuronal aging. **a** Representative western blot images of α-synuclein in Triton X-100-soluble (Tx-sol) and Triton X-100-insoluble (Tx-insol) fractions and in culture media (Media). Asterisks indicate nonspecific antibody binding. The arrowhead indicates the quantified α-synuclein. Quantification of α-synuclein in Tx-sol (**b**), Tx-insol (**c**), and media (**d**). **e**–**h** Inhibition of p21 ameliorated senescence-induced secretion of α-synuclein. **e** Representative western blot images of α-synuclein in Tx-sol, Tx-insol, and media. Asterisks indicate nonspecific antibody binding. The arrowhead indicates the quantified α-synuclein. Quantification of α-synuclein in Tx-sol (**f**), Tx-insol (**g**), and media (**h**). The levels of α-synuclein in cell extracts and media were normalized to the values of β-actin and secretogranin II (SGII), respectively (*n* = 3). **i**, **j** Effects of UC2288 (p21 inhibitor) on the cell-to-cell propagation of α-synuclein. BiFC-positive cells are indicated with arrowheads. Scale bar: 20 µm. **j** Quantification of BiFC-positive cells (*n* = 3, minimum 500 cells per experiment). **k**–**n** Inhibition of p21 reduced TNF-α-induced secretion of α-synuclein. **k** Representative western blot images of α-synuclein in the Tx-sol and Tx-insol fractions. The arrowhead indicates the quantified α-synuclein in Tx-sol. The bar on the right side of the blot indicates the quantified area in Tx-insol. Quantification of α-synuclein in the Tx-sol (**l**) and Tx-insol (**m**) fractions. **n** Secreted α-synuclein, measured by ELISAs. All data are presented as the mean ± SEM (*P < 0.05, ***P < 0.0005, ****P < 0.0001). Statistical significance was determined by one-way ANOVA with Dunnett’s post hoc comparison between groups (**b**–**d**), two-way ANOVA with Tukey’s post hoc comparison between groups (**f**–**h**, **l**–**n**), or two-tailed unpaired Student’s *t* test (**j**).

To examine the role of cellular senescence in the secretion of α-synuclein, we measured the levels of secreted α-synuclein during the in vitro aging of rat primary neurons. During aging, prenatal rat cortical neurons showed an increase in distinct features of senescent cells, including an increase in SA-β-gal activity, nuclear localization of p21^CIP/WAF1^, induction of SASP components, and accumulation of lysosomal contents (Supplementary Fig. 1). α-Synuclein levels in neurons were increased during neuronal aging, an increase that was apparent at 7 days in vitro (DIV7) in the detergent-soluble fraction and later (DIV14) in both the detergent-soluble and detergent-insoluble fractions. In contrast, secretion of α-synuclein by senescent neurons was increased only at DIV14 (Fig. 4a–d).

In the next set of experiments, we investigated whether p21^CIP/WAF1^-dependent cellular senescence signaling regulates the enhanced secretion of α-synuclein in aged neurons. Aged neurons were treated at DIV7 and DIV14 with either vehicle or the p21^CIP/WAF1^ inhibitor UC2288, and cellular levels (detergent-soluble and detergent-insoluble cell extracts) and secretion (culture media) of α-synuclein were assessed. Although treatment with UC2288 did not significantly change the intracellular levels of α-synuclein in neurons at DIV7 or DIV14 (Fig. 4e–g), it reduced the secretion of α-synuclein at DIV14 by 50% (Fig. 4e, h). To evaluate the effect of cellular senescence on the propagation of α-synuclein, we cultured the dual-cell BiFC assay system in the absence or presence of UC2288. We found that UC2288 treatment significantly decreased the number of BiFC-positive cells, indicative of reduced cell-to-cell propagation (Fig. 4i, j). These results suggest that p21^CIP/WAF1^ enhances the senescence-associated secretion of α-synuclein in aged neurons, thereby increasing α-synuclein secretion and propagation.

Next, we investigated the role of the p21^CIP/WAF1^ senescence pathway in TNF-α-induced α-synuclein secretion. To this end, we treated primary neurons with UC2288 or vehicle in the presence or absence of TNF-α. Treatment with UC2288 alone did not significantly change the intracellular levels of α-synuclein (Fig. 4k–m); however, it did completely eliminate the TNF-α-induced increase in α-synuclein secretion (Fig. 4n). These results suggest that α-synuclein is secreted through a p21^CIP/WAF1^-dependent SASP pathway during neuronal senescence and that TNF-α promotes α-synuclein secretion through activation of this neuronal senescence pathway.

### The SASP is mediated by lysosomal exocytosis

**Fig. 5:**
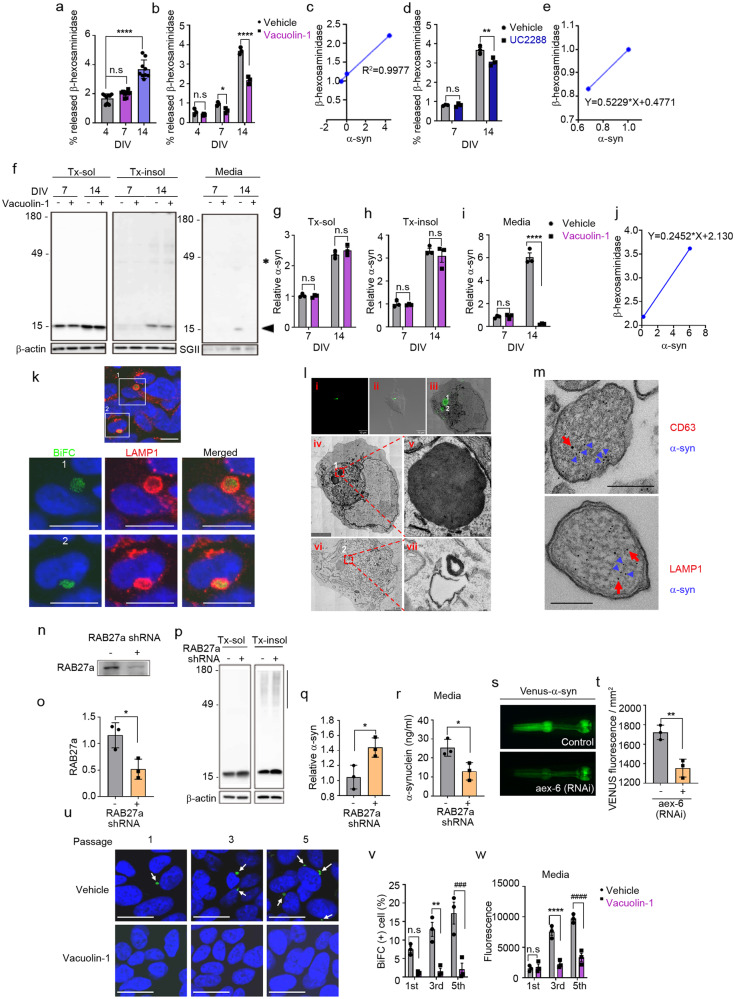
Senescence-associated secretion of α-synuclein is regulated by lysosomal exocytosis. **a** Levels of secreted β-hexosaminidase during neuronal senescence (*n* = 9). **b** Effects of vacuolin-1 on the senescence-induced secretion of β-hexosaminidase (*n* = 3). **c** Correlation between α-synuclein and β-hexosaminidase secretion during senescence (*n* = 3). **d** Effects of UC2288 (p21 inhibitor) on senescence-induced secretion of β-hexosaminidase (*n* = 3). **e** Correlation between α-synuclein and β-hexosaminidase secretion in the presence or absence of UC2288 (*n* = 3). **f**–**i** Inhibition of lysosomal exocytosis reduced senescence-induced secretion of α-synuclein. **f** Representative western blot images of α-synuclein in the Triton X-100-soluble (Tx-sol) fraction, Triton X-100-insoluble (Tx-insol) fraction, and culture media (Media). The arrowhead indicates the quantified synuclein. Asterisks indicate nonspecific antibody binding. Quantification of α-synuclein in Tx-sol (**g**), Tx-insol (**h**), and media (**i**). **j** Correlation between α-synuclein and β-hexosaminidase secretion in the presence or absence of vacuolein-1 (*n* = 3). **k** Subcellular localization of α-synuclein. Green, BiFC-positive α-synuclein aggregates; red, LAMP1; blue, nuclei. Scale bar: 20 μm. Lower panels 1 & 2: magnification of areas bounded by squares in upper panels. **l** CLEM analysis of BiFC-positive structures. Lower panels iv & vi: magnifications of the two BiFC puncta (1 and 2) in Panels i, ii & iii, respectively. Scale bar: 4 μm. Panels v & vii: magnifications of BiFC puncta 1 and 2. **m** Immunoelectron microscopy analysis of α-synuclein-positive lysosome-like structures. Blue arrowheads indicate α-synuclein; red arrows indicate CD63 (upper panel) and LAMP1 (lower panel). Scale bar: 200 nm. **n**–**r** Effects of RAB27a knockdown on α-synuclein secretion. **n**, **o** RAB27a knockdown efficiency. Quantification of RAB 27a in (**o**) (*n* = 3). **p** Representative western blot images of α-synuclein in the Tx-sol and Tx-insol fractions. The bar on the right side of the blot indicates the quantified area in the Tx-insol fraction. **q** Quantification of α-synuclein in the Tx-insol fraction (*n* = 3). **r** Quantification of α-synuclein in culture media by ELISAs (*n* = 3). **s**, **t** Effect of RNAi-mediated RAB27a knockdown on α-synuclein propagation in *C. elegans*. **s** Venus fluorescence in the pharynx, representing the extent of cell-to-cell propagation of α-synuclein in this *C. elegans* model. Quantification of Venus fluorescence at Day 2. More than fifty worms for each line were analyzed (*n* = 3). **u**–**w** Effects of vacuolin-1 on α-synuclein propagation. **u** Representative images of BiFC in passages 1, 3, and 5 in the presence or absence of vacuolin-1. Arrows indicate BiFC puncta. Scale bar: 20 μm. **v** Quantification of the BiFC-positive cells in (**u**) (*n* = 3; minimum 1000 cells per experiment). **w** Quantification of BiFC fluorescence (secreted α-synuclein) in culture media (*n* = 3). The levels of α-synuclein and RAB27a in cell extracts were normalized to those of β-actin, and the levels of proteins in media were normalized to those of secretogranin II (SGII). All data are presented as the mean ± SEM (*P < 0.05, **P < 0.005 ***P < 0.0005, ^###^*P* < 0.0005, ****P < 0.0001, ^####^*P* < 0.0001). Statistical significance was determined by one-way ANOVA with Dunnett’s post hoc comparison between groups (**a**), two-way ANOVA with Tukey’s post hoc comparison between groups (**b**, **d**, **g**–**i**, **v**, **w**), or two-tailed unpaired Student’s *t* test (**o**, **q**, **r**, **t**).

The SASP reflects the non-cell-autonomous ability to generate a rich secretome for communication with the extracellular environment under stress conditions such as cellular aging^39^. Lysosomal exocytosis has been shown to be an unconventional exocytosis pathway known to interact with senescence-related intracellular signaling and is responsible for the secretion of several SASP components^40,41^. These previous studies prompted us to investigate whether in vitro neuronal aging also influences lysosomal exocytosis, evaluated by measuring secreted β-hexosaminidase, a lysosomal enzyme, in the culture medium. Aged neurons at DIV14 showed a significant increase in the levels of secreted β-hexosaminidase compared with younger (DIV4 and 7) neurons (Fig. 5a). Treatment with vacuolin-1, a small-molecule inhibitor of Ca^2+^-dependent lysosomal exocytosis, reduced the secretion of β-hexosaminidase at DIV7 and DIV14 without affecting secretion in young DIV4 neurons (Fig. 5b). The levels of secreted α-synuclein showed a strong positive correlation with those of secreted β-hexosaminidase during neuronal aging (Fig. 5c). Furthermore, addition of the p21^CIP/WAF1^ inhibitor UC2288 reduced the secretion of β-hexosaminidase in neurons at DIV14 but not at DIV7 (Fig. 5d), confirming that lysosomal exocytosis is associated with the SASP. Again, α-synuclein secretion showed a positive correlation with β-hexosaminidase secretion in the presence or absence of UC2288 (Fig. 5e). These findings suggest that SASP is mediated, at least in part, by lysosomal exocytosis.

To determine the role of lysosomal exocytosis in the age-related secretion of α-synuclein, we cultured neurons in the presence or absence of vacuolin-1. Inhibition of lysosomal exocytosis did not significantly affect the intracellular levels of α-synuclein (Fig. 5f–h). In contrast, secretion of α-synuclein was strongly blocked by vacuolin-1 in aged neurons (Fig. 5f, i), suggesting that α-synuclein secretion is mediated by lysosomal exocytosis in aging neurons. There was a strong correlation between α-synuclein secretion and β-hexosaminidase secretion in the presence and absence of vacuolin-1 (Fig. 5j)

To further evaluate the contribution of lysosomal exocytosis to the secretion of α-synuclein, we investigated the subcellular localization of the α-synuclein BiFC signal in the propagation assay system, which indicates the participation of α-synuclein multimers in successive rounds of cell-to-cell propagation^21^. BiFC fluorescence colocalized with LAMP1, a lysosomal membrane protein (Fig. 5k), suggesting that α-synuclein multimers en route to secretion are localized in lysosomes. To further validate the lysosomal localization, we performed correlative light-electron microscopy (CLEM) and found that BiFC fluorescence was localized in multilamellar, electron-dense structures (Fig. 5l). Furthermore, immunoelectron microscopy showed that α-synuclein-positive vesicles were immunoreactive for the lysosomal markers CD63 and LAMP1 (Fig. 5m). In a further test of the role of lysosomal exocytosis in the secretion of α-synuclein, we analyzed α-synuclein secretion following knockdown of human *RAB27a*, which regulates the fusion of lysosome-related organelles with plasma membranes during lysosomal exocytosis. Using shRNA, we achieved a 55% reduction in RAB27a expression (Fig. 5n, o). Under these conditions, α-synuclein secretion was significantly reduced, whereas the levels of detergent-insoluble intracellular α-synuclein were increased (Fig. 5p–r).

We next investigated the role of RAB27a in α-synuclein propagation in the *Caenorhabditis elegans* BiFC model, in which α-synuclein propagation was assessed by monitoring Venus BiFC fluorescence in pharyngeal muscles and neurons^21^. RNAi-mediated knockdown of *aex-6*, an ortholog of human *RAB27a*, significantly reduced BiFC fluorescence (Fig. 5s, t), confirming the role of lysosomal exocytosis in α-synuclein propagation. Consistent with these findings, upon subculturing of the dual-cell BiFC assay system in the presence of vauolin-1, both the number of BiFC-positive cells and the release of BiFC fluorescence into the culture media were dramatically reduced at all passages (Fig. 5u–w). These results suggest that α-synuclein secretion and propagation are mediated by lysosomal exocytosis during neuronal senescence.

### TNF-α promotes the SASP through lysosomal exocytosis

**Fig. 6:**
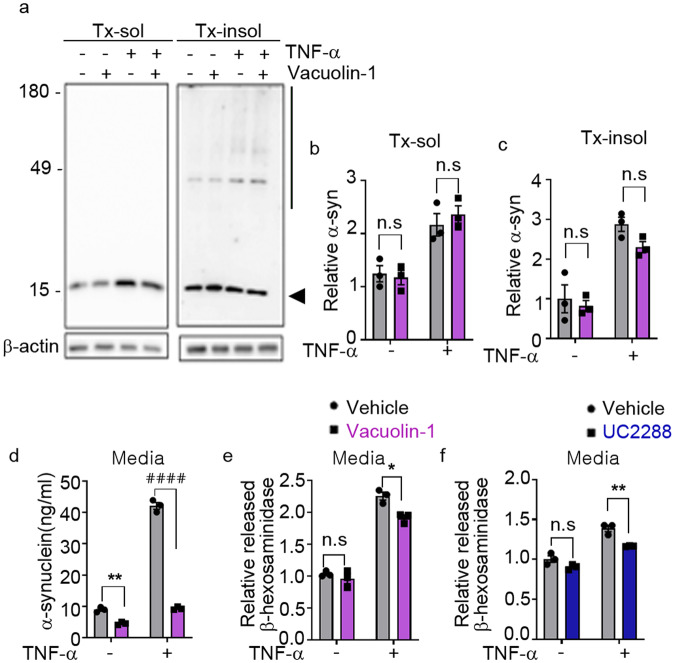
TNF-α-induced SASP mediates secretion of α-synuclein through lysosomal exocytosis. **a**–**d** Inhibition of lysosomal exocytosis reduced TNF-α-induced secretion of α-synuclein. **a** Representative western blot images of α-synuclein in Triton X-100-soluble (Tx-sol) and Triton X-100-soluble-insoluble (Tx-insol) fractions. The arrowhead indicates the quantified α-synuclein in Tx-sol. The line on the right side of the blot indicates the quantified area in Tx-insol. Quantification of α-synuclein in the Tx-sol (**b**) and Tx-insol (**c**) fractions. **d** Secreted α-synuclein, measured by ELISAs. **e**, **f** Effects of vacuolin-1 (**e**) and UC2288 (**f**) on TNF-α-induced secretion of β-hexosaminidase. All data are expressed as the mean ± SEM (*P < 0.05, **P < 0.005, ^####^*P* < 0.0001). Statistical significance was determined by two-way ANOVA with Tukey’s post hoc comparison between groups (**b**–**f**).

Finally, we examined the role of lysosomal exocytosis in TNF-α-induced secretion of α-synuclein. Blockade of lysosomal exocytosis in neurons with vacuolin-1 eliminated the TNF-α-induced secretion of α-synuclein without affecting intracellular α-synuclein levels (Fig. 6a–d). TNF-α treatment also increased the secretion of β-hexosaminidase, an increase that was reversed by vacuolin-1 treatment (Fig. 6e). TNF-α-induced secretion of β-hexosaminidase was also inhibited by treatment with the p21^CIP/WAF1^ inhibitor UC2288 (Fig. 6f), confirming that TNF-α-induced lysosomal exocytosis is associated with neuronal senescence. These results suggest that TNF-α enhances the senescence-associated secretion of α-synuclein through stimulation of lysosomal exocytosis.

## Discussion

Inflammation is an important modulator of protein aggregation and propagation. Here, we show that the inflammatory cytokine TNF-α promotes the cell-to-cell propagation of α-synuclein both in vitro and in vivo. When applied to neuronal cells, TNF-α triggered cellular senescence and acquisition of the SASP. As part of the SASP, α-synuclein secretion was elevated in senescent neurons, an effect that was mediated by lysosomal exocytosis. Taken together, these results demonstrate the relevance of TNF-α in the pathogenesis of PD through the induction of neuronal senescence and acquisition of the SASP and provide clear evidence for the contribution of lysosomal exocytosis to SASP and the secretion and propagation of α-synuclein.

Previous pathological studies have reported evidence of cellular senescence in neurons and glia in neurodegenerative diseases^42^. Features of cellular senescence, including elevated SA-β-gal activity, p53, and DNA damage response (DDR) signals, are detected in brains from individuals with AD and PD^42^. p21^CIP/WAF1^ has also been shown to accumulate in brains from idiopathic PD patients and ischemic stroke donors, as measured in brain slices. These results indicate that cellular senescence in the brain may play a critical role in the pathogenesis of neurodegenerative diseases.

One of the molecular features of senescent cells is the upregulated expression of secretory proteins that constitute the SASP. The SASP, which has also been described as the senescence-messaging secretome, enhances intercellular interactions through autocrine and paracrine signaling, thereby altering the tissue microenvironment^43^. SASP components are elevated in the brains of idiopathic PD patients^44^. In the current study, we showed that neurons exposed to TNF-α display an increase in senescent phenotypes and secretion of α-synuclein through the SASP, suggesting that α-synuclein aggregates can act as SASP components in response to a neuroinflammatory microenvironment. Although the SASP is regarded as a key factor in the regulation of the senescence process, the mechanism underlying secretion in the SASP is still unclear. Some SASP components that lack a signal sequence can be released via various secretory pathways, including the constitutive secretory pathway, recycling pathway, and nonclassical secretory pathway^45–47^. IL-6 is secreted by recycling endosomes^46^, and TNF-α is released via the phagocytic cup^48^. Some SASP components, including IL-1β and IL-18, are localized in secretory lysosomes and released via lysosomal exocytosis^49^. Given that high mobility group box 1 (HMGB1), another major SASP factor, is also found in endolysosome-related organelles^50^, the lysosome-mediated secretory pathway may be a principal route for the secretion of SASP components. This result is consistent with our current finding that α-synuclein is localized to LAMP1/CD63-positive organelles and released via RAB27a-mediated lysosomal exocytosis. Consistent with our results, a previous study showed that disruption of lysosomal exocytosis impairs α-synuclein secretion in neurons^51^. Taken together, these observations indicate that the secretion of SASP components, including α-synuclein aggregates, is mediated by lysosomal exocytosis.

Another example of pathogenic protein aggregates being secreted through lysosomal exocytosis is truncated mutant forms of tau, a process that is regulated in a mucolipin TRP cation channel 1 (TRPML1)-dependent manner by transcription factor EB (TFEB), a master gene in the lysosomal gene network^52^. This finding suggests that lysosomal exocytosis is responsible for the secretion—and perhaps propagation—of tau proteins. Consistent with this observation, our present results demonstrated upregulation of TFEB expression in senescent neurons in association with an increase in α-synuclein secretion (Supplementary Table 1). Furthermore, both basal and TNF-α-induced secretion of α-synuclein were inhibited by genetic or pharmacological blockade of lysosomal exocytosis. Based on previous findings and our current findings, we propose that lysosomal exocytosis is a pivotal mechanism for the secretion of pathological proteins and thus for cell-to-cell propagation of these proteins.

Several studies have suggested that activation of the inflammatory network contributes to the initiation, amplification, and maintenance of cellular senescence^53^. IL-6, which is considered a central regulator and rate-limiting component of the inflammatory network, is known to mediate oncogene-induced senescence^54^. Signaling via the chemokine receptor CXCR2 enhances cellular senescence by activating senescence effectors such as p14 and p15 and the p53/p21 circuit^53^. IL-1β induces cellular senescence by upregulating p16^INK4a^ expression in chondrocytes and astrocytes^55,56^. Long-term exposure or overexpression of TNF-α leads to cellular senescence in various cell types, including fibroblasts, melanoma cells, and hematopoietic cells^57–59^. Our finding that neurons exposed to TNF-α display several features of the senescent phenotype is also consistent with previous studies that collectively indicate a role for inflammatory factors in inducing cellular senescence. Senescent cells also generate proinflammatory signals, such as IL-6, IL-8, and CCL family members^60^. Thus, inflammation and cellular senescence can promote each other, creating a microenvironment that enhances disease pathogenesis.

**Fig. 7:**
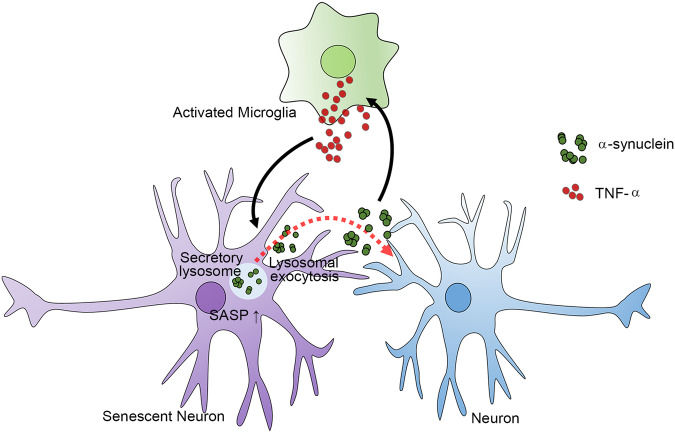
The SASP of neurons induced by TNF-α regulates α-synuclein propagation through lysosomal exocytosis. Activated microglia secrete soluble factors, including TNF-α, which induce neuronal senescence. The SASP of senescent neurons increases α-synuclein secretion through lysosomal exocytosis. The α-synuclein thus secreted is not only transferred to connected neurons as part of cell-to-cell aggregate propagation but also activates glial cells to sustain inflammatory responses.

On the basis of our findings, we propose a model for how glial inflammation, neuronal senescence, and α-synuclein aggregation interact with one another to generate a microenvironment that prolongs PD pathogenesis. In the nervous system, inflammatory activation of microglia increases the production of TNF-α, which then induces neuronal senescence. Senescent neurons produce α-synuclein aggregates and activate SASP through lysosomal exocytosis, which mediates secretion of α-synuclein aggregates and enables their propagation to connected neurons. Secreted α-synuclein can in turn activate microglia and astrocytes, which sustain inflammatory responses. Therefore, inflammation and α-synuclein aggregation promote each other in a feed-forward cycle, and neuronal senescence is a pivotal mediator connecting glial inflammation and neuronal α-synuclein aggregation (Fig. 7).

In conclusion, TNF-α induces neuronal senescence and enhances the SASP of neurons and subsequent lysosome-dependent secretion of α-synuclein aggregates, resulting in increased cell-to-cell propagation of α-synuclein. These findings provide critical insights into the mechanism governing the interplay between neuroinflammation and protein aggregation in disease progression in PD.

### Supplementary information

Supplementary information

## Data Availability

Raw sequencing reads have been deposited at the National Center for Biotechnology Information under BioProject PRJNA797925. The source data are provided with this paper. Other data are available from the corresponding author upon reasonable request.

## References

[1] Schulz-Schaeffer WJ (2010). The synaptic pathology of alpha-synuclein aggregation in dementia with Lewy bodies, Parkinson’s disease and Parkinson’s disease dementia. Acta Neuropathol..

[2] Polymeropoulos MH (1997). Mutation in the alpha-synuclein gene identified in families with Parkinson’s disease. Science.

[3] Singleton AB (2003). alpha-Synuclein locus triplication causes Parkinson’s disease. Science.

[4] Simon-Sanchez J (2009). Genome-wide association study reveals genetic risk underlying Parkinson’s disease. Nat. Genet..

[5] Poewe W (2008). Non-motor symptoms in Parkinson’s disease. Eur. J. Neurol..

[6] Recasens A, Dehay B (2014). Alpha-synuclein spreading in Parkinson’s disease. Front. Neuroanat..

[7] Visanji NP, Brooks PL, Hazrati LN, Lang AE (2013). The prion hypothesis in Parkinson’s disease: Braak to the future. Acta Neuropathol. Commun..

[8] Braak H (2003). Staging of brain pathology related to sporadic Parkinson’s disease. Neurobiol. Aging.

[9] Braak H, Rub U, Jansen Steur EN, Del Tredici K, de Vos RA (2005). Cognitive status correlates with neuropathologic stage in Parkinson disease. Neurology.

[10] Brundin P, Melki R (2017). Prying into the prion hypothesis for Parkinson’s disease. J. Neurosci..

[11] Lee HJ, Bae EJ, Lee SJ (2014). Extracellular alpha-synuclein-a novel and crucial factor in Lewy body diseases. Nat. Rev. Neurol..

[12] Abounit S (2016). Tunneling nanotubes spread fibrillar alpha-synuclein by intercellular trafficking of lysosomes. EMBO J..

[13] Jang A (2010). Non-classical exocytosis of alpha-synuclein is sensitive to folding states and promoted under stress conditions. J. Neurochem..

[14] Bae EJ (2013). Lipid peroxidation product 4-hydroxy-2-nonenal promotes seeding-capable oligomer formation and cell-to-cell transfer of alpha-synuclein. Antioxid. Redox Signal..

[15] Lee HJ (2013). Autophagic failure promotes the exocytosis and intercellular transfer of alpha-synuclein. Exp. Mol. Med..

[16] Kouli A, Camacho M, Allinson K, Williams-Gray CH (2020). Neuroinflammation and protein pathology in Parkinson’s disease dementia. Acta Neuropathol. Commun..

[17] Wang Q, Liu Y, Zhou J (2015). Neuroinflammation in Parkinson’s disease and its potential as therapeutic target. Transl. Neurodegener..

[18] von Herrmann KM (2018). NLRP3 expression in mesencephalic neurons and characterization of a rare NLRP3 polymorphism associated with decreased risk of Parkinson’s disease. NPJ Parkinsons Dis..

[19] Gao HM (2011). Neuroinflammation and alpha-synuclein dysfunction potentiate each other, driving chronic progression of neurodegeneration in a mouse model of Parkinson’s disease. Environ. Health Perspect..

[20] Olanow CW, Savolainen M, Chu Y, Halliday GM, Kordower JH (2019). Temporal evolution of microglia and alpha-synuclein accumulation following foetal grafting in Parkinson’s disease. Brain.

[21] Bae EJ (2014). Glucocerebrosidase depletion enhances cell-to-cell transmission of alpha-synuclein. Nat. Commun..

[22] Lee HJ, Suk JE, Bae EJ, Lee SJ (2008). Clearance and deposition of extracellular alpha-synuclein aggregates in microglia. Biochem. Biophys. Res. Commun..

[23] Lee HJ, Khoshaghideh F, Patel S, Lee SJ (2004). Clearance of alpha-synuclein oligomeric intermediates via the lysosomal degradation pathway. J. Neurosci..

[24] Kim DK (2016). Anti-aging treatments slow propagation of synucleinopathy by restoring lysosomal function. Autophagy.

[25] Kamath RS (2003). Systematic functional analysis of the *Caenorhabditis elegans* genome using RNAi. Nature.

[26] Love MI, Huber W, Anders S (2014). Moderated estimation of fold change and dispersion for RNA-seq data with DESeq2. Genome Biol..

[27] Bindea G (2009). ClueGO: a Cytoscape plug-in to decipher functionally grouped gene ontology and pathway annotation networks. Bioinformatics.

[28] Huang da W, Sherman BT, Lempicki RA (2009). Systematic and integrative analysis of large gene lists using DAVID bioinformatics resources. Nat. Protoc..

[29] Lee HJ (2011). Enzyme-linked immunosorbent assays for alpha-synuclein with species and multimeric state specificities. J. Neurosci. Methods.

[30] Atsaves V, Leventaki V, Rassidakis GZ, Claret FX (2019). AP-1 transcription factors as regulators of immune responses in cancer. Cancers.

[31] Roman J, Ritzenthaler JD, Fenton MJ, Roser S, Schuyler W (2000). Transcriptional regulation of the human interleukin 1beta gene by fibronectin: role of protein kinase C and activator protein 1 (AP-1). Cytokine.

[32] Ogawa K (2002). Transcriptional regulation of the IL-5 gene in peripheral T cells of asthmatic patients. Clin. Exp. Immunol..

[33] Schraml BU (2009). The AP-1 transcription factor Batf controls T(H)17 differentiation. Nature.

[34] Zhu C, Gagnidze K, Gemberling JH, Plevy SE (2001). Characterization of an activation protein-1-binding site in the murine interleukin-12 p40 promoter: demonstration of novel functional elements by a reductionist approach. J. Biol. Chem..

[35] Wang ZY (2005). Regulation of IL-10 gene expression in Th2 cells by Jun proteins. J. Immunol..

[36] Daman AW, Josefowicz SZ (2021). Epigenetic and transcriptional control of interferon-beta. J. Exp. Med..

[37] Kumari R, Jat P (2021). Mechanisms of cellular senescence: cell cycle arrest and senescence associated secretory phenotype. Front. Cell. Dev. Biol..

[38] Moreno-Blas D (2019). Cortical neurons develop a senescence-like phenotype promoted by dysfunctional autophagy. Aging.

[39] Birch J, Gil J (2020). Senescence and the SASP: many therapeutic avenues. Genes Dev..

[40] Carmona-Gutierrez D, Hughes AL, Madeo F, Ruckenstuhl C (2016). The crucial impact of lysosomes in aging and longevity. Ageing Res. Rev..

[41] Ge W, Li D, Gao Y, Cao X (2015). The roles of lysosomes in inflammation and autoimmune diseases. Int. Rev. Immunol..

[42] Martinez-Cue C, Rueda N (2020). Cellular senescence in neurodegenerative diseases. Front. Cell. Neurosci..

[43] Herranz N, Gil J (2018). Mechanisms and functions of cellular senescence. J. Clin. Invest..

[44] Chinta SJ (2018). Cellular senescence is induced by the environmental neurotoxin paraquat and contributes to neuropathology linked to Parkinson’s disease. Cell Rep..

[45] Murray RZ, Stow JL (2014). Cytokine secretion in macrophages: SNAREs, Rabs, and membrane trafficking. Front. Immunol..

[46] Manderson AP, Kay JG, Hammond LA, Brown DL, Stow JL (2007). Subcompartments of the macrophage recycling endosome direct the differential secretion of IL-6 and TNFalpha. J. Cell Biol..

[47] Dinarello CA (2009). Immunological and inflammatory functions of the interleukin-1 family. Annu. Rev. Immunol..

[48] Kay JG, Murray RZ, Pagan JK, Stow JL (2006). Cytokine secretion via cholesterol-rich lipid raft-associated SNAREs at the phagocytic cup. J. Biol. Chem..

[49] Carta S, Lavieri R, Rubartelli A (2013). Different members of the IL-1 family come out in different ways: DAMPs vs. cytokines?. Front. Immunol..

[50] Gardella S (2002). The nuclear protein HMGB1 is secreted by monocytes via a non-classical, vesicle-mediated secretory pathway. EMBO Rep..

[51] Tsunemi T (2019). Increased lysosomal exocytosis induced by lysosomal Ca(2+) channel agonists protects human dopaminergic neurons from alpha-synuclein toxicity. J. Neurosci..

[52] Xu Y (2021). TFEB regulates lysosomal exocytosis of tau and its loss of function exacerbates tau pathology and spreading. Mol. Psychiatry.

[53] Ren JL, Pan JS, Lu YP, Sun P, Han J (2009). Inflammatory signaling and cellular senescence. Cell. Signal..

[54] Kuilman T (2008). Oncogene-induced senescence relayed by an interleukin-dependent inflammatory network. Cell.

[55] Philipot D (2014). p16INK4a and its regulator miR-24 link senescence and chondrocyte terminal differentiation-associated matrix remodeling in osteoarthritis. Arthritis Res. Ther..

[56] Shang D, Hong Y, Xie W, Tu Z, Xu J (2020). Interleukin-1beta drives cellular senescence of rat astrocytes induced by oligomerized amyloid beta peptide and oxidative stress. Front. Neurol..

[57] Mavrogonatou E, Konstantinou A, Kletsas D (2018). Long-term exposure to TNF-alpha leads human skin fibroblasts to a p38 MAPK- and ROS-mediated premature senescence. Biogerontology.

[58] Tyciakova S, Valova V, Svitkova B, Matuskova M (2021). Overexpression of TNFalpha induces senescence, autophagy and mitochondrial dysfunctions in melanoma cells. BMC Cancer.

[59] Beyne-Rauzy O (2004). Tumor necrosis factor alpha induces senescence and chromosomal instability in human leukemic cells. Oncogene.

[60] Davalos AR, Coppe JP, Campisi J, Desprez PY (2010). Senescent cells as a source of inflammatory factors for tumor progression. Cancer Metastasis Rev..

