# Correction: TNF-α promotes α-synuclein propagation through stimulation of senescence-associated lysosomal exocytosis

**DOI:** 10.1038/s12276-024-01215-0

**Published:** 2024-04-01

**Authors:** Eun-Jin Bae, Minsun Choi, Jeong Tae Kim, Dong-Kyu Kim, Min Kyo Jung, Changyoun Kim, Tae-Kyung Kim, Jun Sung Lee, Byung Chul Jung, Soo Jean Shin, Ka Hyun Rhee, Seung-Jae Lee

**Affiliations:** 1 Neuroscience Research Institute, Seoul National University College of Medicine, Seoul, 03080 Korea; 2 Department of Biomedical Sciences, Seoul National University College of Medicine, Seoul, 03080 Republic of Korea; 3 Neural Circuits Research Group, Korea Brain Research Institute, Daegu, 41068 Korea; 4 Department of Exercise Physiology and Sport Science Institute, Korea National Sport University, Seoul, 05541 Republic of Korea; 5 Present Address: Center for Convergence Research of Neurological Disorders, Ajou University School of Medicine, Suwon, 16499 Korea; 6 Present Address: Molecular Neuropathology Section, Laboratory of Neurogenetics, National Institute on Aging, National Institutes of Health, Bethesda, MD 20892 USA; 7 Present Address: Neuramedy Co., Ltd., Seoul, Korea; 8 Present Address: Nutritional Sciences and Toxicology Department, University of California Berkeley, Berkeley, CA 94720 USA

**Keywords:** Parkinson's disease, Neurodegeneration, Cellular neuroscience

Correction to: *Experimental & Molecular Medicine* 10.1038/s12276-022-00789-x, published online 5 July 2022

The authors noticed an error in the Results section after this article was published online. A mistake was made in Fig. 2a and c of the original publication “TNF-α promotes α-synuclein propagation through stimulation of senescence-associated lysosomal exocytosis”. The authors regret to notice that the representative image of Fig. 2a was unintentionally duplicated during figure assembly from Fig. 2c. For Fig. 2, the authors provided updated version. The authors believe the correction would not have any effect on the results of the study or scientific conclusions.
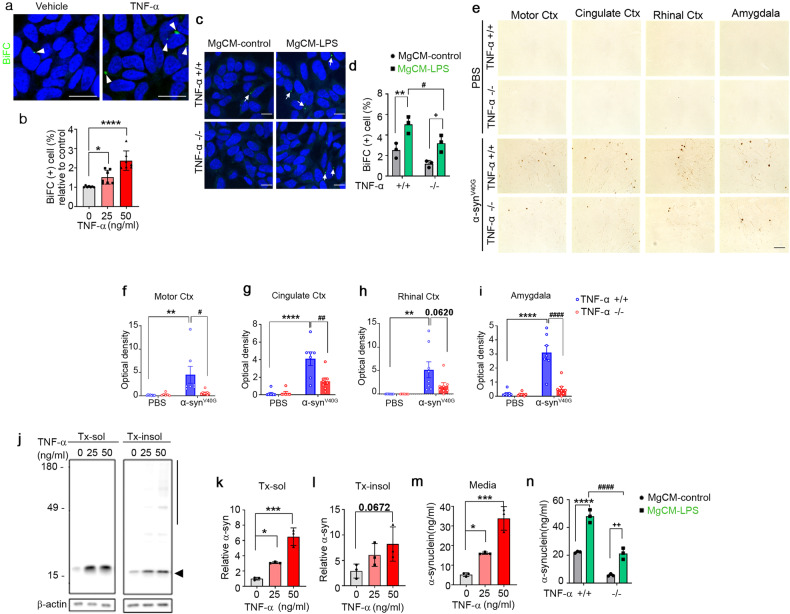


The authors apologize for any inconvenience caused.

The original article has been corrected.

