# Supplementary material for: TNF-α promotes α-synuclein propagation through stimulation of senescence-associated lysosomal exocytosis
Source: Exp Mol Med. 2022 Jul 5;54(6):788–800. doi: 10.1038/s12276-022-00789-x (PMC9352737; doi:10.1038/s12276-022-00789-x)
Supplement: Supplementary file 1 — Supplementary information [file 12276_2022_789_MOESM1_ESM.pdf]

TNF-α promotes α-synuclein propagation through stimulation of senescence-associated lysosomal exocytosis

Eun-Jin Bae[1,2,#], Minsun Choi[2,#], Jeong Tae Kim[2], Dong-Kyu Kim[1,2], Min Kyo Jung[3], Changyoun Kim[1,2], Tae-Kyung Kim[1,2,4], Jun Sung Lee[1,2], Byung Chul Jung[1,2], Soo Jean Shin[2], Ka Hyun Rhee[2], Seung-Jae Lee[1,2*]

[1]Neuroscience Research Institute, Seoul National University College of Medicine, Seoul 03080, Korea

[2]Department of Biomedical Sciences, Seoul National University College of Medicine, Seoul, 03080, Republic of Korea

[3] Neural Circuits Research Group, Korea Brain Research Institute, Daegu 41068, Korea

[4]Department of Exercise Physiology and Sport Science Institute, Korea National Sport University, Seoul, 05541, Republic of Korea

*Corresponding author: Department of Biomedical Sciences, Seoul National University College of Medicine, 103 Daehak-ro, Jongro-gu, Seoul 03080, Korea, Tel: +82-2-3668-7037, Fax: +82-2-447-5683, Emails: sjlee66@snu.ac.kr

[#]These authors contributed equally to this work.

Present Address: Dong-Kyu Kim, Center for Convergence Research of Neurological Disorders, Ajou University School of Medicine, Suwon, 16499, Korea; Changyoun Kim, Molecular Neuropathology Section, Laboratory of Neurogenetics, National Institute on Aging,

National Institutes of Health, Bethesda, MD 20892, USA; Jun Sung Lee, Neuramedy Co. Ltd, Seoul, Korea; Byung Chul Jung, Nutritional Sciences and Toxicology Department, University of California Berkeley, Berkeley, CA 94720

Running title: TNF-α promotes α-synuclein propagation

Supplementary information. Supplementary Figures, legends, and Supplementary tables

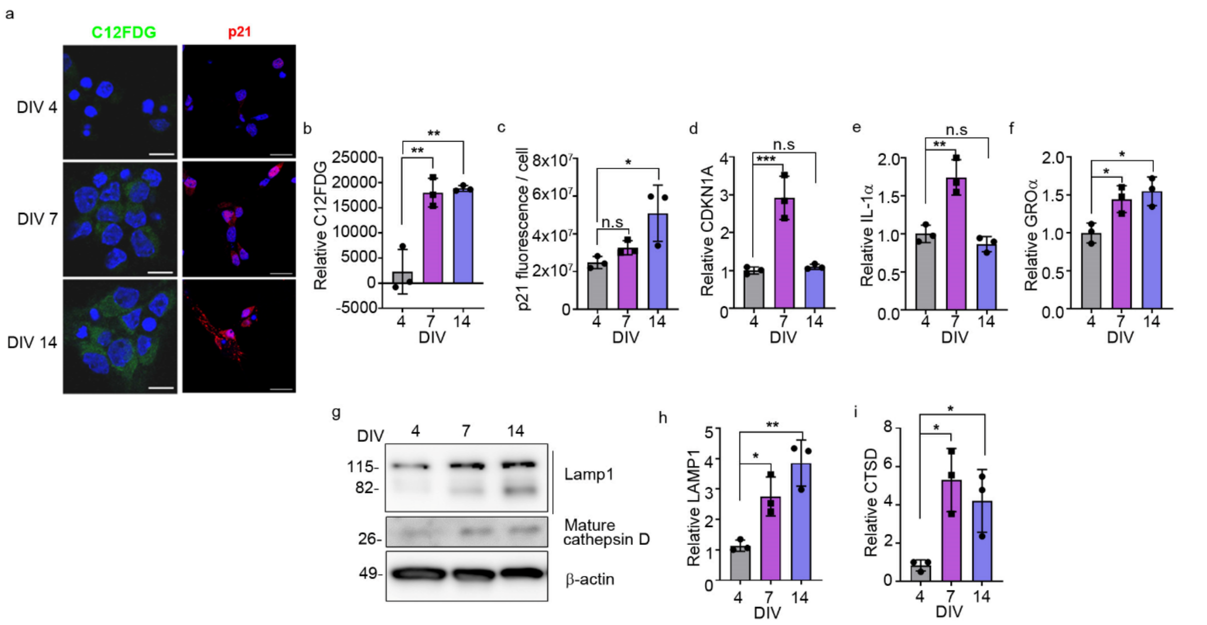

Supplementary Fig. 1. Characterization of neuronal senescence. a Representative images of SA-β-gal (C12FDG) and p21$^{CIP/WAF1}$ in neurons at DIV 4, 7, 14. Green: C12FDG, Red: p21$^{CIP/WAF1}$, Blue: Nuclei, scale bar: 20 μm. **b, c** Quantification of SA-βgal (**b**) and p21$^{CIP/WAF1}$ (**c**). *n*=3, minimum 200 cells per experiment. **d-f** Relative expression of CDKN1A (**d**), IL-1α (**e**), and GROα (**f**) in neurons at DIV 4, 7, 14. *n*=3. **g-i** Lysosomal accumulation in aged neurons. **g** Western blotting images of LAMP1, mature cathepsin D, β-actin. **h, i** Relative level of LAMP1 (**h**), and Cathepsin D (CTSD) (**i**). *n*=3. Statistical significance was determined by one-way ANOVA with Dunnett's post hoc comparison between groups, *P<0.05, **P<0.005, ***P<0.0005. All data are presented as the mean ± SEM.

Supplementary Table 1. The list of differentially expressed genes (DEGs)

Log2 [fold change] ≥ 0.3, adjusted $p < 0.05$

Entrez ID	genesymbol	baseMean	log2 FoldChange	lfcSE	stat	pvalue	padj
692203	SNORD88B	69.07249	-24.2333	3.026687	-8.00655	1.17973E-15	4.63634E-13
63973	NEUROG2	9.700814	-1.85404	0.491228	-3.7743	0.000160457	0.015098805
3488	IGFBP5	20.60321	-1.52786	0.334286	-4.57052	4.86516E-06	0.000670188
56944	OLFML3	26.60472	-1.26763	0.286839	-4.41931	9.90165E-06	0.001236504
4239	MFAP4	54.51322	-0.9236	0.180746	-5.10991	3.2231E-07	5.52143E-05
9260	PDLIM7	68.51838	-0.82483	0.15934	-5.17658	2.25994E-07	4.02106E-05
1191	CLU	112.128	-0.732	0.118597	-6.17216	6.73652E-10	1.72055E-07
3671	ISLR	110.2298	-0.70894	0.120758	-5.87077	4.33783E-09	1.03504E-06
4237	MFAP2	132.8394	-0.62061	0.109763	-5.65407	1.56689E-08	3.52038E-06
10381	TUBB3	279.1793	-0.58427	0.070583	-8.27777	1.25503E-16	5.24052E-14
1281	COL3A1	128.5351	-0.56472	0.10687	-5.28418	1.2627E-07	2.34337E-05
158763	ARHGAP36	373.0038	-0.5538	0.060117	-9.21204	3.19972E-20	1.78144E-17
7431	VIM	360.31	-0.55308	0.062215	-8.88978	6.12282E-19	2.9219E-16
64943	NT5DC2	127.5383	-0.53172	0.107999	-4.92338	8.50602E-07	0.000129156
7846	TUBA1A	711.5104	-0.52464	0.042957	-12.2131	2.647E-34	2.7207E-31
1809	DPYSL3	227.17	-0.52379	0.075999	-6.89205	5.49941E-12	1.56347E-09
3959	LGALS3BP	167.0814	0.383907	0.099302	3.866056	0.00011061	0.010709887
9520	NPEPPS	109.0519	0.411984	0.116145	3.547138	0.000389441	0.031347641
1647	GADD45A	114.255	0.444658	0.11408	3.897778	9.70794E-05	0.009550966
1592	CYP26A1	164.1793	0.446383	0.099685	4.477947	7.53643E-06	0.000970618
11117	EMILIN1	107.5476	0.469916	0.126032	3.728532	0.000192599	0.017714083
3301	DNAJA1	230.2171	0.481216	0.07455	6.454931	1.08269E-10	2.95242E-08
7425	VGF	183.789	0.49803	0.088902	5.601992	2.11903E-08	4.64171E-06
3956	LGALS1	1162.649	0.520731	0.070997	7.334502	2.22548E-13	7.25289E-11
4081	MAB21L1	98.05077	0.580417	0.12424	4.671735	2.98667E-06	0.000429116
1026	CDKN1A	307.8591	0.623353	0.065527	9.512937	1.85353E-21	1.12577E-18
4673	NAP1L1	91.75325	0.637929	0.13249	4.814906	1.47269E-06	0.000218646
2879	GPX4	271.9388	0.644694	0.077609	8.306962	9.81837E-17	4.23203E-14
3672	ITGA1	66.23745	0.657776	0.160953	4.086758	4.37443E-05	0.004752121
1382	CRABP2	3950.209	0.662059	0.050682	13.06304	5.3548E-39	7.15508E-36
5327	PLAT	345.4926	0.697478	0.063885	10.91771	9.48572E-28	7.45577E-25
140606	SELM	124.1633	0.745315	0.11712	6.363664	1.96997E-10	5.26456E-08
54674	LRRN3	48.3279	0.752938	0.197654	3.809371	0.000139321	0.013392859

7157	TP53	54.72948	0.773804	0.180727	4.281611	1.85545E-05	0.002194028
1113	CHGA	39.42116	0.79741	0.222545	3.583139	0.00033949	0.02871054
9686	VGLL4	76.94432	0.797914	0.145979	5.465952	4.60429E-08	8.9163E-06
2907	GRINA	70.64089	0.815382	0.157582	5.174348	2.28708E-07	4.02106E-05
4256	MGP	38.22185	0.823794	0.227602	3.619452	0.000295228	0.025450558
5979	RET	440.1319	0.828268	0.055619	14.89184	3.72351E-50	7.10765E-47
5720	PSME1	124.0667	0.828608	0.110325	7.510635	5.88411E-14	2.01599E-11
3107	HLA-C	43.72144	0.8649	0.214313	4.035679	5.44446E-05	0.005728262
9518	GDF15	191.8001	0.940968	0.091395	10.29559	7.37669E-25	5.47597E-22
148753	FAM163A	48.22715	1.024049	0.197133	5.1947	2.0505E-07	3.70255E-05
6622	SNCA	7758.954	1.029442	0.043112	23.8784	5.1355E-126	3.4311E-122
2335	FN1	136.181	1.047939	0.106838	9.808659	1.03332E-22	6.57489E-20
4793	NFKBIB	25.61581	1.086976	0.28626	3.797161	0.000146363	0.013969291
27233	SULT1C4	32.72069	1.122467	0.249276	4.502909	6.70294E-06	0.000886779
6772	STAT1	22.31443	1.129734	0.309678	3.648098	0.000264189	0.023533945
3572	IL6ST	30.72138	1.181798	0.262622	4.499989	6.79571E-06	0.000890238
10801	SEPT9	153.3012	1.217733	0.102529	11.87699	1.55872E-32	1.48769E-29
100874110	GLYCTK-AS1	35.8213	1.231643	0.253622	4.856211	1.19653E-06	0.000179642
11182	SLC2A6	20.21265	1.278798	0.328111	3.89745	9.72109E-05	0.009550966
3105	HLA-A	157.6995	1.302414	0.101621	12.8164	1.32713E-37	1.6121E-34
7942	TFEB	23.1145	1.323486	0.310216	4.266332	1.98713E-05	0.002329125
10318	TNIP1	54.73554	1.353175	0.190916	7.087809	1.36252E-12	3.95783E-10
5721	PSME2	79.3488	1.409914	0.154282	9.138546	6.32974E-20	3.38312E-17
2669	GEM	32.02186	1.445715	0.261434	5.52994	3.2034E-08	6.48543E-06
9636	ISG15	71.83829	1.645858	0.198008	8.312078	9.40402E-17	4.18855E-14
10133	OPTN	44.33032	1.670252	0.221006	7.557496	4.10903E-14	1.48391E-11
3429	IFI27	12.60598	1.679296	0.463854	3.62031	0.00029425	0.025450558
3726	JUNB	42.82846	1.696475	0.226862	7.478002	7.5461E-14	2.52077E-11
8878	SQSTM1	146.6936	1.773672	0.114229	15.52729	2.26747E-54	6.05958E-51
7980	TFPI2	16.01148	1.8381	0.392345	4.684903	2.80093E-06	0.000411275
9308	CD83	8.105981	1.914151	0.537518	3.56109	0.000369319	0.030275066
92610	TIFA	10.50784	1.917228	0.479752	3.99629	6.43429E-05	0.006649779
6892	TAPBP	58.94462	1.93815	0.195156	9.9313	3.04264E-23	2.03279E-20
64091	POPDC2	8.305981	1.958517	0.536257	3.652201	0.000260003	0.023316486
629	CFB	11.50791	2.001504	0.465557	4.299164	1.71443E-05	0.002045381
4794	NFKBIE	7.505569	2.001587	0.560159	3.573248	0.000352581	0.029328588
4147	MATN2	9.906875	2.07555	0.506865	4.094877	4.22393E-05	0.004626238

4502	MT2A	9.406944	2.079585	0.531612	3.911847	9.1593E-05	0.009133327
1294	COL7A1	14.50976	2.129228	0.42858	4.968102	6.76113E-07	0.000103842
10410	IFITM3	64.64847	2.208646	0.194282	11.36823	6.01939E-30	5.02695E-27
567	B2M	280.4217	2.319822	0.085638	27.08881	1.3339E-161	1.7824E-157
22822	PHLDA1	22.91822	2.377118	0.355472	6.687212	2.27462E-11	6.33197E-09
91107	TRIM47	5.704676	2.41667	0.680412	3.551776	0.000382641	0.031134094
684	BST2	41.22895	2.444423	0.263436	9.279007	1.71066E-20	9.93818E-18
7127	TNFAIP2	7.906602	2.788197	0.622722	4.477433	7.55458E-06	0.000970618
4792	NFKBIA	68.25666	2.872853	0.206875	13.88693	7.60262E-44	1.26983E-40
6891	TAP2	4.403301	2.964964	0.829905	3.572653	0.000353383	0.029328588
972	CD74	15.11272	2.980231	0.483012	6.170104	6.82452E-10	1.72055E-07
8605	PLA2G4C	4.503782	3.001585	0.827062	3.629214	0.000284286	0.025156455
55620	STAP2	4.803782	3.105855	0.825061	3.764393	0.000166954	0.015600276
4791	NFKB2	36.82888	3.162737	0.318437	9.932051	3.01978E-23	2.03279E-20
2537	IFI6	84.2599	3.376919	0.226261	14.92489	2.27012E-50	5.05557E-47
79924	ADM2	5.904745	3.434471	0.807544	4.252984	2.1094E-05	0.002450942
252995	FNDC5	29.22613	3.482744	0.384447	9.059095	1.31537E-19	6.75999E-17
85004	RERG	6.205708	3.5126	0.802547	4.376817	1.20425E-05	0.001466988
4880	NPPC	10.9099	3.659883	0.66504	5.503255	3.72842E-08	7.32635E-06
6236	RRAD	8.207152	3.946417	0.781649	5.048837	4.44507E-07	7.24329E-05
339398	LINGO4	9.509078	4.17157	0.773175	5.395377	6.83797E-08	1.30527E-05
5971	RELB	25.92297	4.364955	0.50048	8.721537	2.7445E-18	1.26455E-15
7185	TRAF1	2.001926	4.444273	1.212171	3.666374	0.000246014	0.022211096
84676	TRIM63	2.001926	4.444273	1.212171	3.666374	0.000246014	0.022211096
8995	TNFSF18	2.202407	4.581916	1.20748	3.794609	0.000147876	0.014013614
51284	TLR7	2.402407	4.707351	1.19519	3.938579	8.19657E-05	0.008360501
4050	LTB	2.602407	4.822745	1.185367	4.068566	4.73033E-05	0.005016401
4940	OAS3	2.602407	4.822745	1.185367	4.068566	4.73033E-05	0.005016401
7124	TNF	2.602407	4.822745	1.185367	4.068566	4.73033E-05	0.005016401
602	BCL3	6.005227	4.826117	1.0628	4.540944	5.60029E-06	0.000748311
148170	CDC42EP5	2.802407	4.929585	1.194538	4.126772	3.6789E-05	0.004130883
3106	HLA-B	2.802889	4.929776	1.17895	4.181498	2.89595E-05	0.003335831
10148	EBI3	3.603852	5.292393	1.156411	4.576566	4.7267E-06	0.000657897
10272	FSTL3	114.2109	5.314509	0.296229	17.94055	5.68938E-72	2.53405E-68
330	BIRC3	3.703852	5.331888	1.149434	4.638709	3.50592E-06	0.000498362
7412	VCAM1	64.36334	5.491202	0.416434	13.18625	1.05294E-39	1.56326E-36
4879	NPPB	24.71871	5.598293	0.720381	7.771294	7.76886E-15	2.96593E-12
6890	TAP1	5.003852	5.765972	1.129625	5.104324	3.31979E-07	5.54488E-05

9536	PTGES	5.305296	5.850309	1.119388	5.226348	1.7289E-07	3.16461E-05
259307	IL4I1	6.405296	6.122181	1.101563	5.557723	2.73317E-08	5.70635E-06
338440	ANO9	7.505778	6.350926	1.091822	5.816817	5.99787E-09	1.40603E-06
8519	IFITM1	8.808666	6.581804	1.067956	6.162992	7.13829E-10	1.76633E-07
29994	BAZ2B	14.61493	7.312244	1.016834	7.191188	6.42299E-13	1.95055E-10
9235	IL32	243.1156	7.919782	0.461304	17.16825	4.59091E-66	1.53359E-62
3383	ICAM1	23.82263	8.017148	0.977157	8.204569	2.3142E-16	9.37043E-14
3604	TNFRSF9	33.33226	8.501731	0.952864	8.922292	4.56735E-19	2.26033E-16
10537	UBD	136.4319	10.53492	0.916766	11.49139	1.4575E-30	1.29834E-27
6347	CCL2	235.633	11.32327	0.914965	12.37563	3.54125E-35	3.94318E-32

Supplementary Table 2. Recombinant cytokines used in figure 1

Name of protein	Manufacturer	Catalog #
Human IL-1β	Prospec	Cat#CYT208
Human IL-2	Prospec	Cat#CYT209
Human IL-4	Prospec	Cat#CYT211
Human IL-5	Prospec	Cat#CYT213
Human IL-6	Prospec	Cat#CHM231
Human IL-8	Prospec	Cat#CHM231
Human IL-10	Prospec	Cat#CYT500
Human IL-12	Prospec	Cat#CYT-101
Human IL-17	Prospec	Cat#CYT250,
Human IFN-α	Prospec	Cat#CYT-520
Human IFN-β	Prospec	Cat#CYT-236
Human IFN-γ	Prospec	Cat#CYT-206
Human TNF-α	Prospec	Cat#CYT223

Supplementary Table 3. Primers used in this study

Gene		Sequence of Primer	Application
Mouse TNF	Forward	CCTCTTCTCATTCCTGCTTGTGG	qRT-PCR
	Reverse	GGTGGTTTGTGAGTGTGAGGG	
Mouse CDKN1A	Forward	CCCGTGGACAGTGAGCAGTT	qRT-PCR
	Reverse	CCAGACGAAGTTGCCCTCCAG	
Mouse TP53	Forward	GTGCTCACCCTGGCTAAAGT	qRT-PCR
	Reverse	AGGAGGATGAGGGCCTGA AT	
Mouse JAK1	Forward	CGTCAA ACCTGTGTCTCG CT	qRT-PCR
	Reverse	CCCCCAAAGTCTACGCTGTT	
Mouse STAT1;	Forward	GATCGCTTGCCCAACTCTTG	qRT-PCR
	Reverse	ACTGTGACATCCTTGGGCTG	
Mouse GROα	Forward	ACTGCACCCAAACCGAAGTC	qRT-PCR
	Reverse	TTTCTGAACCAAGGGAGCTTC A	
Mouse IL-6	Forward	GCCAGAGTCCTTCAGAGAGATACA	qRT-PCR
	Reverse	TGTTAGGAGAGCATTGGA AATTGGG	
Mouse IL-1α	Forward	GCACTTGGGAGCCCTTTCAT	qRT-PCR
	Reverse	ACAGCTTTAAGGACGGGAGG	
Mouse GAPDH	Forward	AGAAGGTCGTGAAGCAGGCATC	qRT-PCR
	Reverse	CGAAGGTGGAAGAGTGGGAGTTG	

Supplementary Table 4. Antibodies used in this study

Name of Antibody	Manufacturer	Catalog #	Dilution used
Mouse monoclonal anti-α-synuclein (Syn-1)	BD Biosciences	Cat#610787	1:1500 (WB)<br>1:250 (ELISA)
Rabbit polyclonal anti-α-synuclein	Cell Signaling Tech	Cat#2642	1:10 (Immuno-EM)
Rabbit monoclonal anti-phospho-α-synuclein (EP1536Y)	Abcam	Cat#ab51253	1:500 (IHC)
Rabbit monoclonal anti-α-synuclein (biotinylated)	Cell Signaling Tech	Cat#74184	1:1500 (ELISA)
Mouse monoclonal anti-LAMP1	Abcam	Cat#ab25630	1:10 (Immuno-EM)
Rabbit polyclonal anti-Lamp1	Abcam	Cat#ab24170	1:1000 (WB)
Mouse monoclonal anti-CD63	BD Biosciences	Cat#556019	1:10 (Immuno-EM)
Mouse monoclonal anti-Cathepsin D	Abcam	Cat#ab6313	1:1000 (WB)
Mouse monoclonal anti-p21	Abcam	Cat#GT1032	1:1000 (IF)
Polyclonal anti-mouse IgG gold secondary antibody	Sigma-Aldrich,	Cat#G7652	1:50 (Immuno-EM)
Polyclonal anti-rabbit IgG gold secondary antibody	Sigma-Aldrich,	Cat#G7277	1:50 (Immuno-EM)
Alexa fluor 488-anti-mouse secondary antibody	Jackson Immunoresearch Laboratories	Cat#115-545-062	1:200 (IF)
HRP-anti-Rabbit secondary antibody	Bio-Rad	Cat#170-6515	1:200 (IHC)
HRP-anti-mouse secondary antibody	Bio-Rad	Cat#170-6516	1:3000 (WB)
